# Supplementary material for: Assessing vector competence of mosquitoes from northeastern France to West Nile virus and Usutu virus
Source: PLoS Negl Trop Dis. 2023 Jun 5;17(6):e0011144. doi: 10.1371/journal.pntd.0011144 (PMC10270612; doi:10.1371/journal.pntd.0011144)
Supplement: S1 Table — The method is described in [45]. One mosquito leg was placed in a tube containing the PCR mix composed of two antisense primers at a final concentration of 0.15 μM, the sense primer at 0.25 μM, buffer (1X), dNTPs at 250 μM, MgCl2 at 1.5 mM, BSA (Bovine serum Albumin) at 0.135μg/μL, one unit of Taq polymerase and 5 μL of the DNA extract. The primers used were: pipCQ11R 5’-CATGTTGAGCTTCGGTGAA-3’, molCQ11R 5’-CCCTCCAGTAAGGTATCAAC-3’ and CQ11F2 5’-GATCCTAGCAAGCGAGAAC-3’. The amplification program started with 15 min at 94°C, 35 cycles of 94°C for 30s, 54°C for 30s and 72°C for 40s and finally a 5 min elongation phase at 72°C. PCR products were separated by electrophoresis on a 2% agarose gel. (PDF) [file pntd.0011144.s001.pdf]

S1 Table. Molecular assay based on indels in the flanking region of a microsatellite locus CQ11 to distinguish the two forms of *Culex pipiens*, *pipiens* and *molestus*. The method is described in (1). One mosquito leg was placed in a tube containing the PCR mix composed of two antisense primers at a final concentration of 0.15 µM, the sense primer at 0.25 µM, buffer (1X), dNTPs at 250 µM, MgCl2 at 1.5 mM, BSA (Bovine serum Albumin) at 0.135µg/µL, one unit of Taq polymerase and 5 µL of the DNA extract. The primers used were: pipCQ11R 5'-CATGTTGAGCTTCGGTGAA-3', molCQ11R 5'-CCCTCCAGTAAGGTATCAAC-3' and CQ11F2 5'-GATCCTAGCAAGCGAGAAC-3'. The amplification program started with 15 min at 94°C, 35 cycles of 94°C for 30s, 54°C for 30s and 72°C for 40s and finally a 5 min elongation phase at 72°C. PCR products were separated by electrophoresis on a 2% agarose gel.

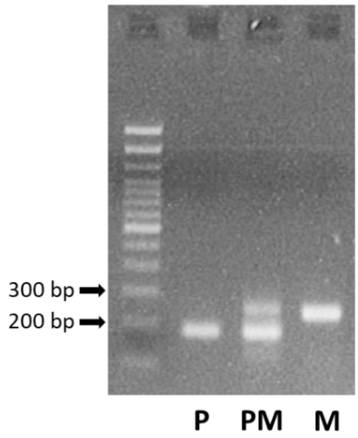

P, *Cx. pipiens pipiens*; M, *Cx. pipiens molestus*; PM, hybrids *pipiens*/*molestus*

**Population**	**Machault**	**Maine**	**Sainte-Croix**	**Verzy**
**Number of mosquitoes tested**	19	16	16	17
**Pipiens**	5.3%	6.25%	87.5%	100%
**Molestus**	52.6%	50%	6.25%	-
**Hybrids**	42.1%	43.75%	6.25%	-

References

1. Bahnck CM, Fonseca DM. Rapid assay to identify the two genetic forms of Culex (Culex) pipiens L. (Diptera: Culicidae) and hybrid populations. The American journal of tropical medicine and hygiene. 2006;75(2):251-5.
